# Impact of the ultra‐long 48 mm drug‐eluting stent on procedural and clinical outcomes in patients with diffuse long coronary artery disease

**DOI:** 10.1002/clc.23997

**Published:** 2023-02-20

**Authors:** Young Jin Youn, Ho Sung Jeon, Young In Kim, Jung‐Hee Lee, Young Jun Park, Dong‐Hyuk Cho, Jung‐Woo Son, Jun‐Won Lee, Min‐Soo Ahn, Sung Gyun Ahn, Jang‐Young Kim, Byung‐Su Yoo, Seung‐Hwan Lee, Junghan Yoon

**Affiliations:** ^1^ Department of Internal Medicine, Division of Cardiology, Wonju Severance Christian Hospital Yonsei University Wonju College of Medicine Wonju South Korea

**Keywords:** clinical outcome, coronary artery disease, drug‐eluting stent

## Abstract

**Background:**

Diffuse coronary artery disease (CAD) is a prognostic factor after percutaneous coronary intervention (PCI) and requires multiple overlapping stent implantations.

**Hypothesis:**

We investigated the impact of ultra‐long 48 mm drug‐eluting stent (DES) on procedural and clinical outcomes in real‐world practice.

**Methods:**

Patients who underwent DES implantation for a lesion length of >40 mm were selected from a prospective registry between 2019 and 2021. Patients treated with one or more ultra‐long 48 mm DES were in the ultra‐long DES group (*n* = 221). The others comprised the conventional DES group (*n* = 428). Procedural and clinical outcomes were compared after propensity score matching (PSM). The primary endpoint was a device‐oriented composite outcome (DOCO) consisting of cardiac death, target vessel‐related myocardial infarction, and target lesion revascularization at 1‐year follow‐up.

**Results:**

After PSM, 158 matched pairs of patients showed no differences in the baseline clinical and angiographic characteristics. The stent delivery failure rate, the use of guide‐extension catheter or anchor balloon technique, and the procedural success rate were similar for both groups. Approximately two‐thirds of lesions could be treated with one DES in the ultra‐long DES group. At 1‐year follow‐up, the DOCO was similar for both groups (2.5% vs. 0.6%, *p* = .168).

**Conclusions:**

In daily clinical practice, ultra‐long DES implantation is as safe and effective as multiple overlapping conventional DES implants in treating diffuse long CAD. However, ultra‐long DES can reduce the number of stents. (Trial Registration: ClinicalTrials.gov Identifier: NCT02038127).

## Graphical abstract

Impact of and ultra‐long drug‐eluting stent on procedural and clinical outcomes for diffuse long coronary artery disease.

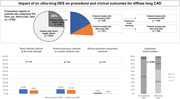

**Abbreviations**
CAD: coronary artery disease
DAPT: dual antiplatelet therapy
DES: drug‐eluting stent
DOCO: device‐oriented composite outcome
IVUS: intravascular ultrasound
MI: myocardial infarction
OCT: optical coherence tomography
PCI: percutaneous coronary intervention
POCO: patient‐oriented composite outcome
RVD: reference vessel diameter
TLR: target lesion revascularization
TVR: target vessel revascularization

## 1 INTRODUCTION

Multiple overlapping stent implantations are usually required for treating diffuse long coronary artery disease (CAD). The current generation of drug‐eluting stents (DESs) revealed excellent safety and efficacy across a broad range of patients and lesion subsets; however, lesion length remains a significant predictor of adverse outcomes, including restenosis and stent thrombosis.^1^, ^2^, ^3^, ^4^ Multiple stents with variable overlap lengths can increase the risk of geographical miss and mechanical complications, such as side‐branch occlusion. In animal models and human intracoronary optical coherence tomography (OCT) imaging, the overlapped DES segment revealed persistent inflammation, fibrin deposition, and delayed endothelization due to local blood flow disturbance and a higher drug/polymer dose.^5^, ^6^, ^7^ Another concern about multiple stent implantation is an increased procedural time, contrast volume, radiation dose, and procedure cost.^8^

Therefore, using ultra‐long 48 mm DES to cover the diseased segment with a minimal number of stents is highly appealing. However, delivering the ultra‐long DES may be challenging, especially in tortuous or severely calcified coronary arteries. Moreover, discrepancies in reference vessel diameters (RVDs) between the proximal and distal landing zones may make selecting the appropriate stent size challenging.

The aim of this study was to evaluate the procedural success and 1‐year safety and efficacy of the ultra‐long DES implantation compared to multiple overlapping conventional DESs in treating diffuse long CAD in real‐world practice.

## 2 METHODS

### 2.1 Study population

This study is a sub‐analysis of the Gangwon percutaneous coronary intervention (PCI) registry (ClinicalTrials.gov Identifier: NCT02038127). Gangwon PCI registry is an unrestricted, prospective, multicenter, and observational registry which enrolled all‐comer patients who underwent PCI from four hospitals. Among patients who underwent PCI between January 2019 and December 2021, as the ultra‐long 48 mm DES was available, we selected patients with at least one lesion length of >40 mm. Patients treated with one or more ultra‐long DESs, either 48 mm Xience Xpedition (everolimus‐eluting stent, Abbott Vascular) or 48 mm Synergy XD (everolimus‐eluting stent, Boston Scientific), comprised the ultra‐long DES group. The others were included in the conventional DES group. The local Institutional Review Board approved the study protocol (CR322080), and this study was conducted according to the principles of the Declaration of Helsinki, revised in 2013. Written informed consent was obtained from all patients.

### 2.2 Procedure

PCI was performed according to the standard technique. Decisions of the vascular access site, pre‐dilatation or direct stenting, the use of glycoprotein IIb/IIIa inhibitors, use of intravascular ultrasound (IVUS) or OCT, use of a pressure wire, a technique for bifurcation or chronic total occlusion lesion, and stent selection including type, length, and diameter was at the operator's discretion. A staged procedure was allowed, which was not counted as revascularization.

The selection and duration of antiplatelet therapy was at the operator's discretion. A loading dose of aspirin (300 mg) and P2Y12 inhibitor (clopidogrel [600 mg], prasugrel [60 mg], or ticagrelor [180 mg]) was administered to all the patients at least 6 h before the procedure unless the patient had been taking these medications or the procedure was an emergency. After the index PCI, dual antiplatelet therapy (DAPT) with a maintenance dose of aspirin (100 mg) and P2Y12 inhibitor (clopidogrel [75 mg QD], prasugrel [10 mg QD], or ticagrelor [90 mg BID]) was encouraged for at least 12 months. Guideline‐directed medical treatment using beta‐blockers, renin‐angiotensin‐aldosterone system inhibitors, and statins was also encouraged.

### 2.3 Data collection and follow‐up

Independent research personnel prospectively collected clinical, angiographic, procedural, and outcome data using a reporting system. Clinical follow‐up was undertaken at 1, 6, and 12 months via office visits, medical record reviews, or telephone interviews. Data regarding patients' clinical status, all interventions, and outcome events were recorded at every visit. However, routine follow‐up angiography was not mandatorily performed.

### 2.4 Study endpoints

The primary endpoint was a device‐oriented composite outcome (DOCO) consisting of cardiac death, myocardial infarction (MI) not clearly attributable to a nontarget vessel, and clinically indicated target lesion revascularization (TLR). The secondary endpoints were PCI success; stent delivery failure at the first attempt; procedural complications; a patient‐oriented composite outcome (POCO) consisting of death, MI, and revascularization; Academic Research Consortium defined stent thrombosis and each component of the primary and secondary endpoints. Details and definitions of endpoints have been previously described.^9^

### 2.5 Statistical analyses

One lesion with a longer lesion length or severe diameter stenosis was selected for lesion analysis in patients treated for multi‐vessel CAD. Target lesion‐ or vessel‐related event was restricted to the selected lesion. Categorical variables are presented as numbers (percentages) and were compared using the *χ* ^2^ test or Fisher's exact test. Continuous variables are presented as mean ± standard deviation or median (interquartile range) and were compared using an independent sample *t*‐test or a Mann–Whitney U test. Cumulative events of clinical outcomes were assessed using Kaplan–Meier estimates and compared with the log‐rank test. All clinical endpoints were analyzed until the date of an endpoint event, loss to follow‐up, or up to 12 months after the index procedure. The propensity scores were estimated with multivariate logistic regression analyses using lesion length, RVD, follow‐up duration, and all covariates listed in Table 1. The propensity scores yielded a C statistic of 0.662, indicating a good ability to differentiate between both groups. Nearest‐neighbor matching with a caliper of 0.005 was used. Statistical analyses were performed using SPSS, version 21.0 (IBM Corp) and SAS, version 9.3 (SAS Institute Inc). A two‐sided *p* < .05 was considered significant.

**Table 1:** Baseline, angiographic, and procedural characteristics in the overall population.

	Conventional DES (*n* = 428)	Ultra‐long DES (*n* = 221)	*p* Value	Standardized difference
Male	310 (72.4)	171 (77.4)	.102	.114
Age, years	68.9 ± 11.2	68.1 ± 10.8	.373	.074
Hypertension	296 (69.2)	142 (64.3)	.216	.104
Diabetes	178 (41.6)	87 (39.4)	.585	.045
Dyslipidemia	196 (45.8)	88 (40.0)	.156	.112
CKD	134 (31.3)	71 (32.1)	.832	.018
Dialysis dependent	13 (3.0)	13 (5.9)	.080	.138
Smoking, Ex‐ or current	265 (61.9)	149 (67.4)	.167	.115
Prior MI	22 (5.1)	20 (9.0)	.055	.153
Prior PCI	60 (14.0)	45 (20.4)	.038	.169
Prior CABG	5 (1.2)	1 (0.5)	.669	.080
Stable angina	57 (13.3)	43 (19.5)	.040	0.166
Unstable angina	121 (28.3)	46 (20.8)	.039	0.174
NSTEMI	133 (31.1)	56 (25.3)	.128	0.128
STEMI	64 (15.0)	41 (18.6)	.238	0.096
Medication at discharge				
Aspirin	426 (99.5)	220 (99.5)	>.999	0.002
P2Y12 inhibitor	428 (100)	221 (100)	n/a	n/a
Clopidogrel	223 (52.1)	107 (48.4)	.254	0.053
Ticagrelor	196 (45.8)	111 (50.2)	.284	0.089
Prasugrel	9 (2.1)	3 (1.4)	.760	.058
Statin	330 (88.8)	195 (88.2)	.835	.017
Calcium channel blocker	51 (11.9)	30 (13.6)	.545	0.050
Beta‐blocker	257 (60.0)	133 (60.2)	.974	0.003
ACEI or ARB	255 (59.6)	132 (59.7)	.971	.003
Multi‐vessel disease	375 (87.6)	188 (85.1)	.364	0.074
ISR lesion	28 (6.5)	20 (9.0)	.247	.094
Tortuosity, moderate to severe	28 (6.5)	22 (10.0)	.122	0.124
Angulation, moderate to extreme	91 (21.3)	49 (22.2)	.789	0.022
Calcification, moderate to severe	180 (42.1)	84 (38.0)	.320	.083
Treated territory			.027	.214
LAD	235 (54.9)	110 (49.8)		
LCX	29 (6.8)	11 (5.0)		
RCA	138 (32.2)	94 (42.5)		
LM involvement	26 (6.1)	6 (2.7)		
Multi‐vessel PCI	203 (47.4)	114 (51.6)	.316	0.083
Elective PCI	356 (83.2)	170 (76.9)	.054	0.157
Transradial access	409 (95.6)	207 (93.7)	.298	0.084
IVUS‐guidance	301 (70.3)	152 (68.8)	.684	0.034
CTO PCI	35 (8.2)	11 (5.0)	.132	0.129
Bifurcation PCI	264 (61.7)	113 (51.1)	.010	0.214
With two‐stent technique	14 (3.3)	4 (1.8)	.238	0.093

*Note*: Data presented as *n* (%) or mean ± standard deviation.

Abbreviations: ACEI, angiotensin converting enzyme inhibitor; ARB, angiotensin II receptor blocker; CABG, coronary artery bypass graft; CKD, chronic kidney disease; DES, drug‐eluting stent; ISR, in‐stent restenosis; IVUS, intravascular ultrasound; LAD, left anterior descending; LCX, left circumflex; LM, left main; MI, myocardial infarction; NSTEMI, non‐ST‐elevation myocardial infarction; PCI, percutaneous coronary intervention; RCA, right coronary artery; STEMI, ST‐elevation myocardial infarction.

## 3 RESULTS

### 3.1 In overall population

Among 2709 patients who underwent PCI, 699 (26%) patients with at least one lesion of length >40 mm were selected, as illustrated in Supporting Information: Figure S1. After excluding patients with cardiogenic shock at presentation or who died during the index hospitalization, 428 patients were treated with the conventional DES, and 221 were treated with at least one ultra‐long DES.

Baseline characteristics, angiographic, and procedural characteristics in the overall population are summarized in Table 1. There were more prior MI, prior PCI, and stable angina but less unstable angina in the ultra‐long DES group than in the conventional DES group. Discharge medications were similar in both groups. There were fewer elective PCI and PCI for the left main coronary artery but more PCI for the right coronary artery and fewer PCI for bifurcation lesions in the ultra‐long DES group than in the conventional DES group.

Procedural outcomes are summarized in Table 2. Despite a similar rate of stent delivery failure at the first attempt and the use of guide‐extension catheter, anchor balloon technique, noncompliant balloon, or scoring balloon, the procedural complication rate was significantly lower in the ultra‐long DES group (17.1% vs. 10.4%, *p* = .024) than in the conventional DES group. Approximately two‐thirds of lesions (68.8%) could be treated with one DES in the ultra‐long DES group. Although the balloon‐to‐vessel ratio was significantly larger in the ultra‐long DES group (0.88 ± 0.13 vs. 0.92 ± 0.11, *p* < .001), stent diameter (3.05 ± 0.38 vs. 2.96 ± 0.36 mm, *p* = .004) was significantly smaller in the ultra‐long DES group. And stent length (62.5 ± 14.4 vs. 58.6 ± 17.5 mm, *p* = .003) was significantly shorter in the ultra‐long DES group than in the conventional DES group. Despite similar fluoroscopic dose and time, the contrast volume (188 ± 51 vs. 174 ± 52 mL, *p* = .001) was significantly lower in the ultra‐long DES group than in the conventional DES group.

**Table 2:** Procedural outcomes.

	Overall population			Propensity‐score‐matched population		
	Conventional DES (*n* = 428)	Ultra‐long DES (*n* = 221)	*p* Value	Conventional DES (*n* = 158)	Ultra‐long DES (*n* = 158)	*p* Value
Implanted stent type			<.001			<.001
One ultra‐long DES	0	152 (68.8)		0	102 (64.6)	
One ultra‐long DES + conventional DES	0	55 (24.9)		0	44 (27.8)	
Overlapped ultra‐long DES	0	13 (5.9)		0	11 (7.0)	
Overlapped ultra‐long DES + conventional DES	0	1 (0.5)		0	1 (0.6)	
Overlapped conventional DES	428 (100)			158 (100)		
Implanted stent number per lesion			<.001			<.001
1	0	152 (68.8)		0 (0)	102 (64.6)	
2	380 (88.8)	63 (28.5)		145 (91.8)	51 (32.3)	
3 or more	48 (11.2)	6 (2.7)		13 (8.2)	5 (3.2)	
Implanted stent diameter per lesion, mm	3.05 ± 0.38	2.96 ± 0.36	.004	3.00 ± 0.36	2.99 ± 0.36	.650
Implanted stent length per lesion, mm	62.5 ± 14.4	58.6 ± 17.5	.003	61.1 ± 12.5	60.5 ± 18.7	.722
Stent delivery failure at the first attempt	58 (13.6)	21 (9.5)	.135	19 (12.0)	14 (8.9)	.358
Rotational atherectomy	14 (3.3)	0 (0.0)	.003	0 (0)	0 (0)	n/a
Guide‐extension catheter use	48 (11.2)	26 (11.8)	.835	18 (11.4)	16 (10.1)	.717
Anchor balloon use	4 (0.9)	0 (0)	.305	1 (0.6)	0 (0)	>.999
Noncompliant balloon use	96 (22.4)	38 (17.2)	.126	32 (20.3)	28 (17.7)	.667
Scoring balloon use	11 (2.6)	3 (1.4)	.314	3 (1.9)	2 (1.3)	.652
Balloon‐to‐vessel ratio	0.88 ± 0.13	0.92 ± 0.11	<.001	0.88 ± 0.14	0.91 ± 0.11	.030
PCI success	420 (98.1)	219 (99.1)	.143	155 (98.1)	156 (98.7)	.623
Procedural complications	73 (17.1)	23 (10.4)	.024	22 (13.9)	18 (11.4)	.499
No‐reflow	25 (5.8)	8 (3.6)	.222	6 (3.8)	7 (4.4)	.777
Side branch occlusion	18 (4.2)	5 (2.3)	.205	7 (4.4)	4 (2.5)	.357
Distal embolization	11 (2.6)	4 (1.8)	.541	5 (3.2)	3 (1.9)	.723
Edge dissection	11 (2.6)	1 (0.5)	.068	4 (2.5)	1 (0.6)	.371
Perforation	1 (0.2)	1(0.5)	>.999	0 (0)	1 (0.6)	>.999
Acute thrombosis	1 (0.2)	0 (0)	>.999	1 (0.6)	0 (0)	>.999
Stent migration	2 (0.5)	0 (0)	.550	1 (0.6)	0 (0)	>.999
Contrast volume, mL	188 ± 51	174 ± 52	.001	185 ± 50	170 ± 52	.012
Fluoroscopic dose, Gy·cm^2^	206.6 ± 120.6	221.5 ± 138.3	.157	199.6 ± 108.6	216.1 ± 144.5	.254
Fluoroscopic time, min	19.6 ± 11.3	19.8 ± 12.2	.893	18.6 ± 9.6	19.0 ± 12.3	.713

*Note*: Data presented as *n* (%) or mean ± standard deviation.

DES, drug‐eluting stent; PCI, percutaneous coronary intervention.

**Figure 1:**
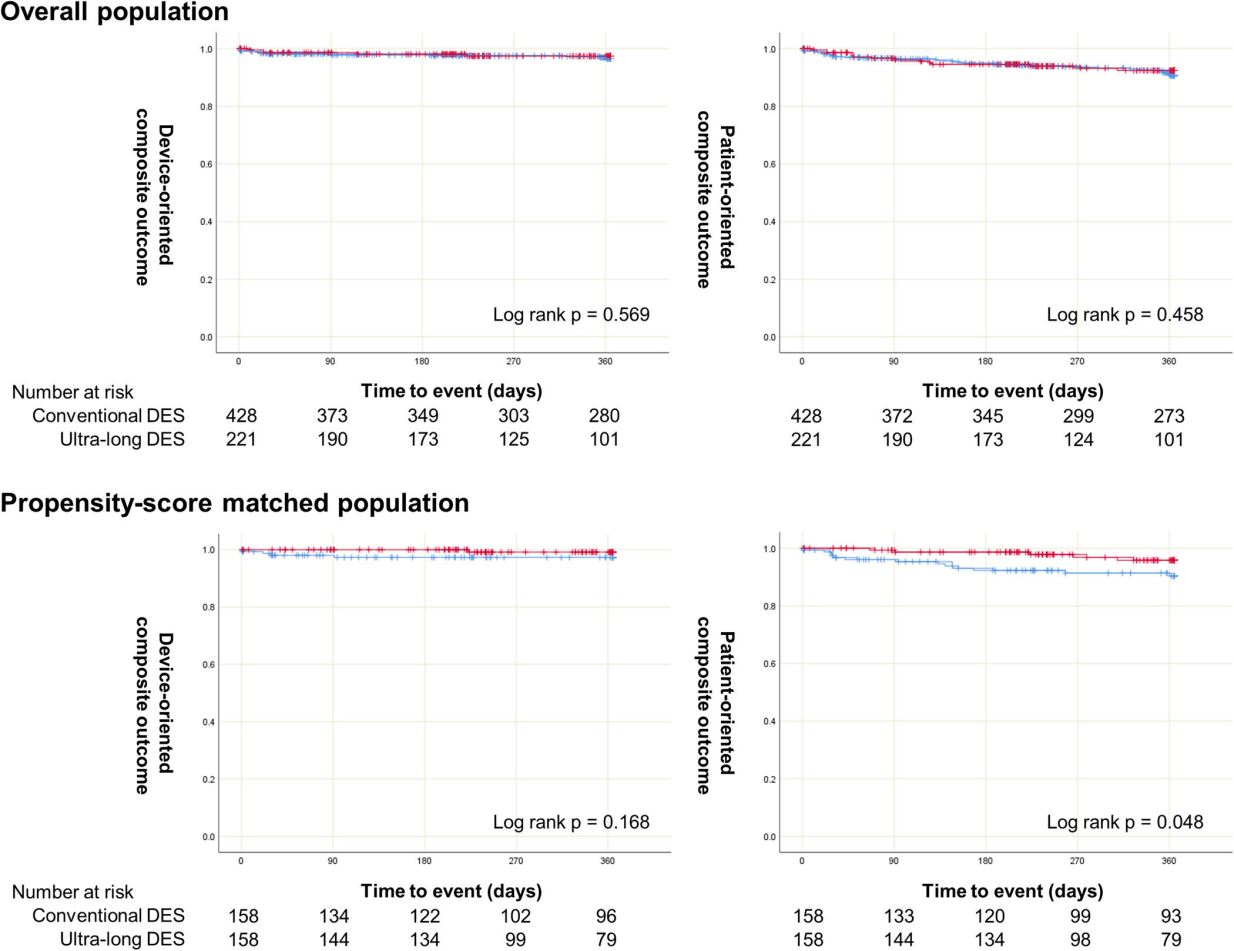
Kaplan–Meier curves for clinical outcomes between the conventional drug‐eluting stent (DES) group (blue line) and the ultra‐long DES group (red line).

Clinical outcomes at 1‐year follow‐up are summarized in Table 3 and Figure 1. The DOCO and POCO were similar in both groups. There was no TLR, target vessel‐related MI, or target vessel revascularization (TVR) event in the ultra‐long DES group.

**Table 3:** Clinical outcomes at 1‐year follow‐up.

	Overall population			Propensity‐score‐matched population		
	Conventional DES (*n* = 428)	Ultra‐long DES (*n* = 221)	Log rank *p* Value	Conventional DES (*n* = 158)	Ultra‐long DES (*n* = 158)	Log rank *p* Value
Follow‐up, days	365 (228, 365)	342 (203, 365)	<.001	365 (201, 365)	355 (221, 365)	.363
Device‐oriented composite outcome	13 (3.0)	5 (2.3)	.569	4 (2.5)	1 (0.6)	.168
Cardiovascular death	4 (0.9)	5 (2.3)	.286	4 (2.5)	1 (0.6)	.168
Target vessel‐related MI	4 (0.9)	0 (0)	.305^a^	0 (0)	0 (0)	n/a
Target lesion revascularization	9 (2.1)	0 (0.0)	.032^a^	0 (0)	0 (0)	n/a
Patient‐oriented composite outcome	34 (7.9)	14 (6.3)	.458	13 (8.2)	5 (3.2)	.048
Any death	16 (3.7)	13 (5.9)	.210	9 (5.7)	4 (2.5)	.139
Any myocardial infarction	8 (1.9)	0 (0)	.056^a^	3 (1.9)	0 (0)	.087^a^
Any revascularization	17 (4.0)	1 (0.5)	.010	3 (1.9)	1 (0.6)	.302
Target vessel revascularization	11 (2.6)	0 (0.0)	.019^a^	1 (0.6)	0 (0)	.297^a^
Definite or probable stent thrombosis	5 (1.2)	1 (0.5)	.669	1 (0.6)	0 (0)	n/a

*Note*: Data presented as *n* (%) or median (interquartile range).

Abbreviations: DES, drug‐eluting stent; MI, myocardial infarction.

^a^ No statistics are computed because all the cases are censored.

### 3.2 In propensity‐score matched population

After propensity‐score matching, the 158 patients in each group were compared. Baseline, angiographic, and procedural characteristics were well‐balanced between both groups (Supporting Information: Table S1).

Procedural outcomes are summarized in Table 2. After adjusting clinical and angiographic characteristics, the rate of stent delivery failure at the first attempt and using a guide‐extension catheter, anchor balloon technique, noncompliant balloon, or scoring balloon remained similar in both groups. In addition, procedural complications were similar in both groups. Although the balloon‐to‐vessel ratio was significantly larger in the ultra‐long DES group (0.88 ± 0.14 vs. 0.91 ± 0.11, *p* = .030), stent diameter (3.00 ± 0.36 vs. 2.99 ± 0.36 mm) was similar between the two groups. Despite the similar total stent length (61.1 vs. 60.5 mm), 64.6% of the lesions could be treated with one DES when the ultra‐long DES was used. Additionally, the contrast volume (185 ± 50 vs. 170 ± 52 mL, *p* = .012) was significantly lower in the ultra‐long DES group than in the conventional DES group.

Clinical outcomes at 1‐year follow‐up are summarized in Table 3 and Figure 1. After the adjustment, both groups had no differences in DOCO, TLR, target vessel‐related MI, and TVR. We provided the detailed cause of death in Supporting Information: Table S2 because the rate of all‐cause death was reversed before and after propensity score matching.

## 4 DISCUSSION

We evaluated the procedural success and 12‐month safety and efficacy of the ultra‐long DES implantation in treating diffuse long CAD in all‐comer patients and compared outcomes with those of multiple overlapping conventional DES implantation. Several findings emerged from our analyses. First, 26% of the patients had a lesion longer than 40 mm and thus were required to be treated with <40 mm multiple DESs. Second, stent deliverability—as regards stent delivery failure at the first attempt and the use of guide‐extension catheter or anchor balloon technique—did not differ between both groups. Third, approximately two‐thirds of lesions could be treated with one ultra‐long DES. Fourth, using the ultra‐long DES could reduce the contrast volume. Finally, device‐related adverse events during the 1‐year follow‐up were few, without statistical differences, regardless of using an ultra‐long DES.

Diffuse CAD is a significant prognostic factor after PCI.^2^, ^3^, ^4^ Multiple overlapping stent implantation has become routine in treating diffuse long CAD^10^, ^11^, ^12^ but may increase (1) the potential for geographical miss, which can be a risk for restenosis or stent thrombosis, (2) difficulty in accessing the side branch owing to stent overlapping at the side branch ostium, which can be a risk for periprocedural MI, and (3) persistent inflammation, fibrin deposition, and delayed endothelization at the overlapped DES segment, which can be a risk for stent thrombosis.^5^, ^6^, ^7^ Therefore, the ultra‐long DES could be an attractive option. Furthermore, the ultra‐long DES might reduce cost, procedure time, contrast volume, and radiation dose.^8^

However, there is potential concern about using the ultra‐long DES. First is a size discrepancy between the proximal and distal RVD. The current ultra‐long DES has the same diameter along the stent length; therefore, appropriate stent‐sizing could be problematic.^13^ Selecting the proximal RVD as the stent diameter may result in over‐expansion at a distal segment and cause a distal edge dissection. On the contrary, selecting the distal RVD may cause under‐expansion at a proximal segment and increase the risk of stent thrombosis. In addition, post‐dilatation for proximal stent apposition might cause a stent fracture, longitudinal deformation, and polymer damage, increasing the risk of stent thrombosis and restenosis.^14^, ^15^ Despite these concerns, meticulous stent deployment can avoid these complications. Following the manufacturer's instructions, the Xience Xpedition with a diameter of ≥3.5 mm and Synergy XD with a diameter of ≥4.0 mm can be dilated up to 5.6 mm and 5.75 mm without polymer deformation, respectively. Thus, the operators should be aware of the maximum permitted overexpansion of these devices. In this study, we could not determine whether the operator chose a proximal or distal RVD for stent‐sizing; nonetheless, the edge dissection rate was similar in both groups.

In this study, we could not find differences in device‐related outcomes between both groups after adjustment. Moreover, there is a relative lack of clinical data supporting whether the longer DES is better than the overlapping DES because extremely long lesions are often under‐represented in clinical trials, and there are no direct comparison trials. However, in the era of newer‐generation DESs, overlapping DES is considered comparable with single DES, with conflicting results.^16^, ^17^, ^18^, ^19^ Animal models suggested that second‐generation DESs may be more suitable to overlap with strut coverage and endothelialization.^20^

In previous studies based on a single‐arm registry, the 48 mm Xience Xpedition showed good safety and efficacy for up to 2 years.^13^, ^21^, ^22^ Sim et al. compared the single 48 mm DES (*n* = 117) over the overlapping DES (*n* = 101) in a nonrandomized, retrospective study. They reported that both strategies revealed comparable clinical outcomes after 2 years; nonetheless, they did not perform any adjustments for confounders.^23^ Hsiao et al. also compared the single 48 mm DES (*n* = 149) with the overlapping DES (*n* = 149) after propensity score‐matching and reported no differences in clinical outcomes between both strategies.^24^ However, they did not report the procedural outcomes. This study is consistent with previous studies as regards clinical outcomes. However, our study included procedural outcomes of both strategies.

Our study revealed low event rates, and there are several reasons. First, patients with cardiogenic shock at presentation or who died during the index hospitalization were excluded; Second, periprocedural MI was not included; Third, routine follow‐up coronary angiography was not mandatory; Finally, a significant proportion of patients underwent PCI using the transradial access and IVUS guidance.

Our study has several limitations. First, it was not a randomized controlled trial; therefore, it has inherent potential bias. After propensity score‐matching, POCO became significant in the conventional DES group. This may reflect that more fragile patients with a more hostile coronary anatomy, where the ultra‐long DES could not cross the lesion, could have been included in the conventional DES group despite a statistical adjustment. Second, the stent choice was at the operator's discretion. The experienced operator might not choose the ultra‐long DES in extremely hostile lesion because the operator already knows that the ultra‐long DES cannot be easily delivered into such a lesion. The higher TLR rate in the conventional DES group among the overall population could be related to lesion characteristics rather than stent selection. Third, statistical power could be low owing to the small sample size and low event rates. Fourth, various stent platforms were used in the conventional DES group. Thus, a direct comparison of stent types was not possible. Finally, a 1‐year follow‐up might be too short; therefore, a longer‐term follow‐up should be considered for a more robust conclusion.

## 5 CONCLUSION

In daily clinical practice, using ultra‐long DES is as safe and effective as multiple overlapping conventional DES implantation in treating diffuse long CAD. In addition, ultra‐long DES can reduce the number of stents and contrast volume. However, larger randomized controlled trials with longer‐term follow‐ups should be performed to draw a definitive conclusion.

## Supporting information

Supporting information.

Supporting information.

## Data Availability

The data that support the findings of this study are available on request from the corresponding author. The data are not publicly available due to privacy or ethical restrictions.
