# Supplementary material for: Impact of the ultra‐long 48 mm drug‐eluting stent on procedural and clinical outcomes in patients with diffuse long coronary artery disease
Source: Clin Cardiol. 2023 Feb 20;46(4):416–24. doi: 10.1002/clc.23997 (PMC10106662; doi:10.1002/clc.23997)
Supplement: Supplementary file 1 — Supporting information. [file CLC-46-416-s001.docx]

**Table S1. Baseline, angiographic, and procedural characteristics in the propensity-score-matched population**.

|  | Conventional DES (n = 158) | Ultra-long DES (n = 158) | *p*-value | Standardized difference |
| --- | --- | --- | --- | --- |
| Male sex | 126 (79.7) | 117 (74.1) | 0.230 | 0.135 |
| Age, years | 68.8 ± 11.0 | 68.4 ± 11.1 | 0.748 | 0.036 |
| Hypertension | 99 (62.7) | 100 (63.3) | 0.907 | 0.013 |
| Diabetes | 65 (41.1) | 57 (36.1) | 0.355 | 0.104 |
| Dyslipidemia | 67 (42.4) | 62 (39.2) | 0.567 | 0.064 |
| CKD | 49 (31.0) | 48 (30.4) | 0.903 | 0.014 |
| Dialysis dependent | 7 (4.4) | 6 (3.8) | 0.777 | 0.032 |
| Smoking, Ex- or current | 110 (69.6) | 100 (63.3) | 0.233 | 0.134 |
| Prior MI | 11 (7.0) | 7 (4.4) | 0.332 | 0.109 |
| Prior PCI | 26 (16.5) | 27 (17.1) | 0.880 | 0.017 |
| Prior CABG | 2 (1.3) | 1 (0.6) | >0.999 | 0.065 |
| Stable angina | 29 (18.4) | 25 (15.8) | 0.550 | 0.067 |
| Unstable angina | 37 (23.4) | 35 (22.2) | 0.789 | 0.030 |
| NSTEMI | 44 (27.8) | 51 (32.3) | 0.390 | 0.097 |
| STEMI | 24 (15.2) | 26 (16.5) | 0.758 | 0.035 |
| Medication at discharge |  |  |  |  |
| Aspirin | 158 (100) | 157 (99.4) | >0.999 | 0.113 |
| P2Y12 inhibitor | 158 (100) | 158 (100) | n/a | n/a |
| Clopidogrel | 79 (50.0) | 74 (46.8) | 0.556 | 0.063 |
| Ticagrelor | 4 (2.5) | 2 (1.3) | 0.431 | 0.089 |
| Prasugrel | 75 (47.5) | 82 (51.9) | 0.684 | 0.093 |
| Statin | 141 (89.2) | 142 (89.9) | 0.854 | 0.021 |
| Calcium channel blocker | 21 (13.3) | 24 (15.2) | 0.629 | 0.054 |
| Beta-blocker | 90 (57.0) | 97 (61.4) | 0.423 | 0.090 |
| ACEI or ARB | 92 (58.2) | 96 (60.8) | 0.647 | 0.052 |
| Multi-vessel disease | 131 (82.9) | 137 (86.7) | 0.347 | 0.106 |
| ISR lesion | 11 (7.0) | 15 (9.5) | 0.413 | 0.092 |
| Tortuosity, moderate to severe | 11 (7.0) | 11 (8.9) | 0.532 | 0.070 |
| Angulation, moderate to extreme | 32 (20.3) | 34 (21.5) | 0.782 | 0.031 |
| Calcification, moderate to severe | 64 (40.5) | 58 (36.7) | 0.488 | 0.078 |
| Treated territory |  |  | 0.174 | 0.004 |
| LAD | 77 (48.7) | 87 (55.1) |  |  |
| LCX | 15 (9.5) | 7 (4.4) |  |  |
| RCA | 55 (34.8) | 58 (58) |  |  |
| LM involvement | 11 (7.0) | 6 (3.8) |  |  |
| Multi-vessel PCI | 71 (44.9) | 76 (48.1) | 0.573 | 0.063 |
| Elective PCI | 129 (81.6) | 123 (77.8) | 0.401 | 0.095 |
| Transradial access | 152 (96.2) | 150 (95.9) | 0.585 | 0.062 |
| IVUS-guidance | 109 (69.0) | 110 (69.6) | 0.903 | 0.014 |
| CTO PCI | 15 (9.5) | 10 (6.3) | 0.297 | 0.117 |
| Bifurcation PCI | 86 (54.4) | 87 (55.1) | 0.910 | 0.013 |
| With two-stent technique | 7 (4.4) | 3 (1.9) | 0.199 | 0.145 |

Data presented as n (%) or mean ± standard deviation.

ACEI = angiotensin converting enzyme inhibitor; ARB = angiotensin II receptor blocker; CABG = coronary artery bypass graft; CKD = chronic kidney disease; DES = drug-eluting stent; ISR = in-stent restenosis; IVUS = intravascular ultrasound; LAD = left anterior descending; LCX = left circumflex; LM = left main; MI = myocardial infarction; NSTEMI = non-ST-elevation myocardial infarction; PCI = percutaneous coronary intervention; RCA = right coronary artery; STEMI = ST-elevation myocardial infarction.

**Table S2. Causes of death**

| **Conventional DES group** | | |  | **Ultra-long DES group** | | |
| --- | --- | --- | --- | --- | --- | --- |
| **Age/sex** | **PSM** | **Death cause** |  | **Age/sex** | **PSM** | **Death cause** |
| **CV death** | | |  | **CV death** | | |
| 74/F | 1 | AMI related cardiogenic shock |  | 81/M | 1 | Stroke related |
| 78/M | 1 | AMI related cardiogenic shock |  |  |  |  |
| 75/M | 1 | Sudden cardiac death |  |  |  |  |
| 69/M | 1 | HF related |  |  |  |  |
|  |  |  |  | 84/M | 0 | AMI related cardiogenic shock |
|  |  |  |  | 80/M | 0 | AMI and fatal arrhythmia |
|  |  |  |  | 70/M | 0 | AMI related |
|  |  |  |  | 62/M | 0 | Stroke related |
| **Non-CV death** | | |  | **Non-CV death** | | |
| 83/M | 1 | Respiratory failure |  | 86/M | 1 | Sepsis |
| 78/M | 1 | Respiratory failure |  | 85/M | 1 | Renal failure |
| 64/M | 1 | Respiratory failure |  | 82/F | 1 | Sepsis |
| 74/F | 1 | Respiratory failure |  |  |  |  |
| 75/F | 1 | Sepsis |  |  |  |  |
| 77/F | 0 | Respiratory failure |  | 78/M | 0 | Respiratory failure |
| 76/F | 0 | Respiratory failure |  | 76/M | 0 | Renal failure |
| 81/F | 0 | Sepsis |  | 73/M | 0 | Renal failure |
| 81/M | 0 | Cancer related |  | 52/M | 0 | Renal failure |
| 75/M | 0 | Renal failure |  | 82/F | 0 | Respiratory failure |
| 71/F | 0 | Renal failure |  |  |  |  |
| 81/F | 0 | Renal failure |  |  |  |  |

DES = drug-eluting stent; AMI = acute myocardial infarction; CV = cardiovascular; HF = heart failure; PSM = propensity score matching.Al
