# Supplementary figures and images for: Impact of the ultra‐long 48 mm drug‐eluting stent on procedural and clinical outcomes in patients with diffuse long coronary artery disease
Source: Clin Cardiol. 2023 Feb 20;46(4):416–24. doi: 10.1002/clc.23997 (PMC10106662; doi:10.1002/clc.23997)

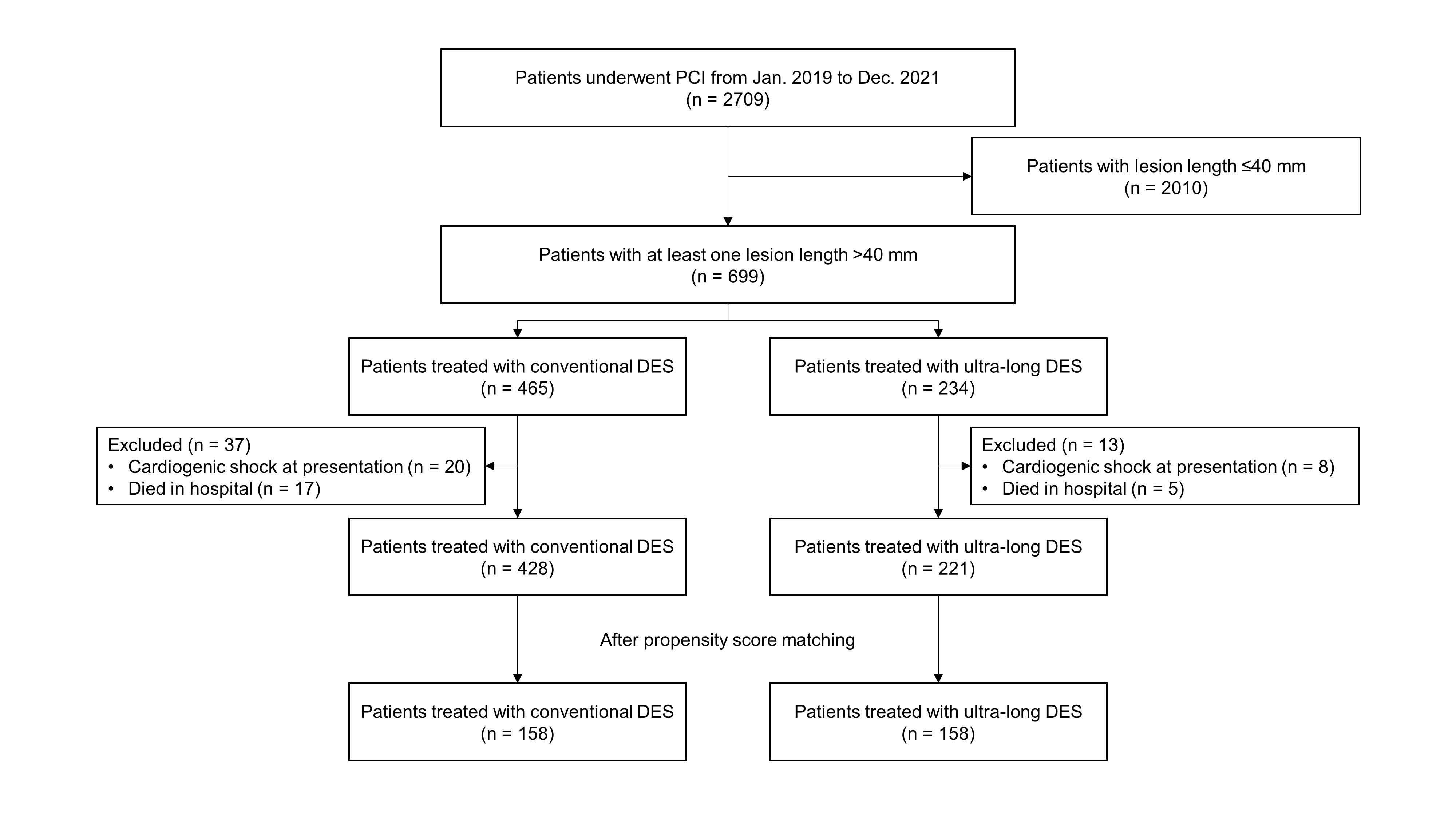

Supplement: Supplementary file 2 — Supporting information. [file CLC-46-416-s002.TIF]
